# Hypermethylation of NDN promotes cell proliferation by activating the Wnt signaling pathway in colorectal cancer

**DOI:** 10.18632/oncotarget.17580

**Published:** 2017-05-03

**Authors:** Yu-Han Hu, Qing Chen, Yan-Xia Lu, Jian-Ming Zhang, Chun Lin, Fan Zhang, Wen-Juan Zhang, Xiao-Min Li, Wei Zhang, Xue-Nong Li

**Affiliations:** ^1^ Department of Pathology, School of Basic Medical Sciences, Southern Medical University, Guangzhou, China; ^2^ Department of Pathology, Xinxiang Medical University, Xinxiang, China; ^3^ Department of Surgery, Nanfang Hospital, Southern Medical University, Guangzhou, China

**Keywords:** NDN, LRP6, Wnt signaling pathway, colorectal cancer, proliferation

## Abstract

The progression of CRC is a multistep process involving several genetic changes or epigenetic modifications. NDN is a member of the MAGE family, encoding a protein that generally suppresses cell proliferation and acting as a transcriptional repressor. Immunohistochemical staining revealed that the expression of NDN was significantly down-regulated in CRC tissues compared with normal tissues and the down-regulation of NDN in CRC could reflect the hypermethylation of the NDN promoter. Treatment of the CRC cell line SW480 with the demethylating agent 5-Aza-CdR restored the NDN expression level. The down-regulation of NDN was closely related to poor differentiation, advanced TNM stage and poor prognosis of CRC. The inhibition of NDN promoted CRC cell proliferation by enriching cells in the S phase. Furthermore, we observed that NDN binds to the GN box in the promoter of LRP6 to attenuate LRP6 transcription and inhibit the Wnt signaling pathway in CRC. In conclusion, our study revealed that the hypermethylation of NDN promotes cell proliferation by activating the Wnt signaling pathway through directly increasing the transcription of LRP6 in CRC. These findings might provide a new theoretical basis for the pathogenesis of CRC and facilitate the development of new therapeutic strategies against CRC.

## INTRODUCTION

Colorectal cancer (CRC) is one of the most common malignancies with high morbidity and mortality [1]. The development of CRC is a complex process characterized by the accumulation of genetic mutations and epigenetic alterations. Mutations on adenomatous polyposis coli (APC), BRAF, TP53 and KRAS have been identified as critical factors for the initiation of CRC [2, 3]. And most of the mutations could result in the activation of the canonical Wnt signaling pathway in CRC. The abnormal activation of the Wnt signaling pathway caused by mutations in these genes has been observed in 85% of sporadic CRC patients [4].

Wnt signaling pathway is a fundamental mechanism during embryonic development and tissue homeostasis. The abnormal activation of the Wnt signaling pathway stimulates the expression of a number of target genes that drive tumorigenesis [5, 6]. When the Wnt signaling pathway is activated, it will result in β-catenin nuclear accumulation and target gene transcription, which is regulated through Frizzled (FZD) receptor and low-density lipoprotein receptor-related protein 5 or 6 (LRP5 or LRP6) co-receptors [7]. And LRP6 promotes cancer cell proliferation and tumorigenesis by altering β-catenin subcellular distribution [8]. Previous studies have demonstrated that the oncogenic KRAS signaling pathway promotes Wnt/β-catenin pathway through the inhibition of GSK3β and the phosphorylation of LRP6 in CRC [9–11]. The results of screening based on ChIP sequencing suggested that LRP6 might be a specific target gene of NDN [12].

Necdin (NDN), a member of the melanoma antigen gene (MAGE) family [13], is a maternally imprinted gene located on chromosome 15q11 that encodes the necdin protein with multiple functions. NDN suppresses cell proliferation in post-mitotic neurons, acting as a transcriptional repressor that recognizes guanosine (G)-rich DNA sequences [14–16]. Recently, accumulating evidence has revealed that NDN is frequently down-regulated in various types of cancers such as urothelial cancer, ovarian cancer, breast cancer and prostate cancer, suggesting that this gene is a potential tumor suppressor [12, 17–19].

Although NDN is down-regulated in many types of cancers, the regulatory mechanism remains unclear. Previous studies have demonstrated that NDN is one of the genes silenced through deletion, maternal uniparental disomy or translocation in Prader-Will syndrome (PWS), and the promoter hypermethylation contributes to the down-regulation of NDN in cancer [12, 17, 18, 20]. The DNA methylation of cytosine bases in CpG dinucleotides plays an important role in cancer development [21], for example, the hypermethylation of CDKN2A, LATS1, TSG and CHD5 in CRC lead to the silencing of these genes and contribute to the promotion of colorectal carcinogenesis [22–24].

Presently, we showed that NDN is down-regulated in CRC, reflecting the hypermethylation of the NDN promoter. In the context of the putative transcriptional regulation of LRP6, we addressed whether NDN/LRP6 plays a modulatory role in the canonical Wnt pathway in CRC.

## RESULTS

### Down-regulation of NDN is correlated with progression and poor prognosis in CRC

Analysis of GEO (http://www.ncbi.nlm.nih.gov/geo/) CRC microarray dataset revealed that NDN was markedly down-regulated in primary CRC tissues as compared with normal colon tissues (*p*<0.001) (Supplementary Figure 1, NCBI/GEO/GSE41258 n=240).

**Figure 1:**
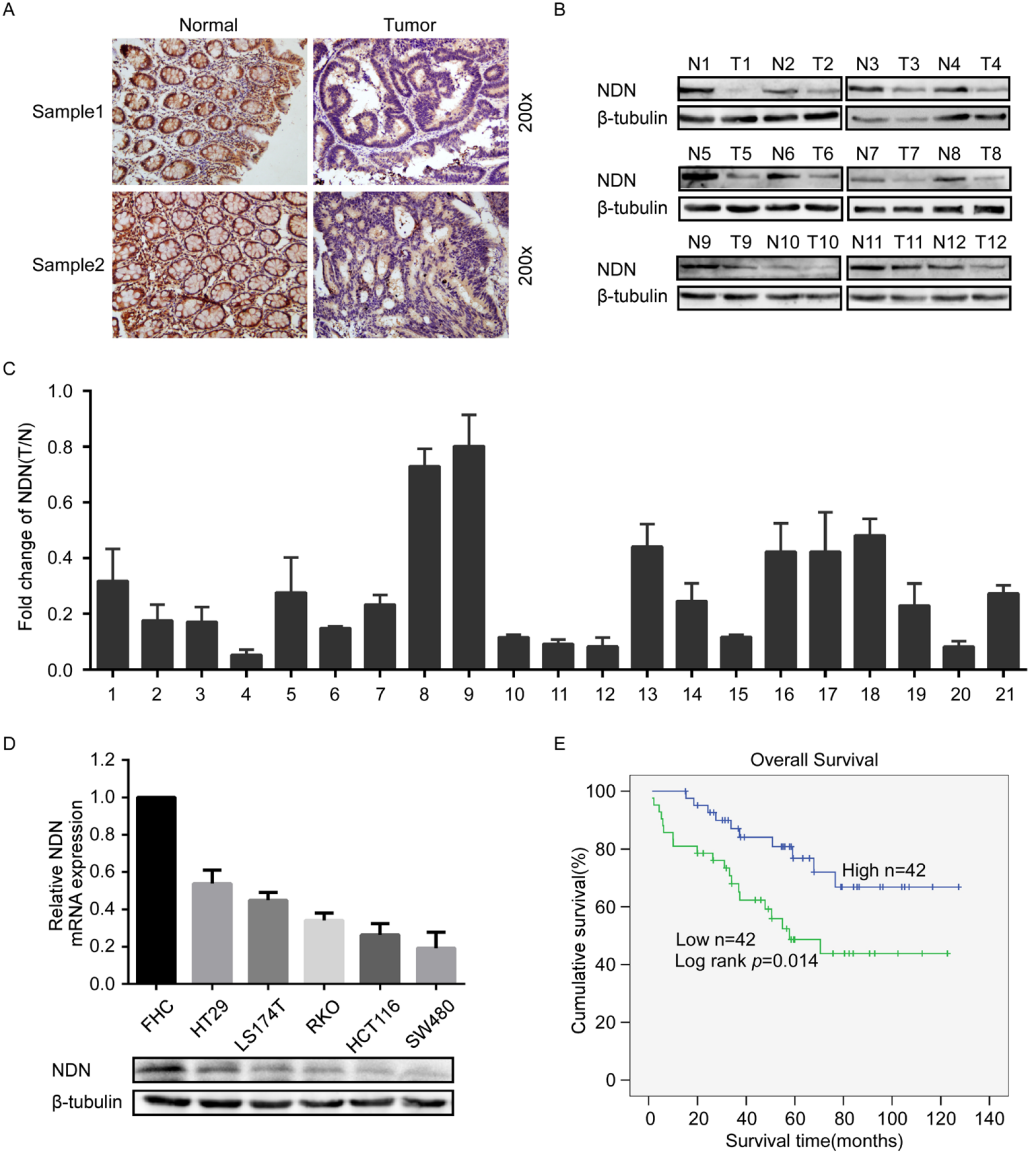
Expression of NDN in CRC and its correlation with CRC prognosis **(A)** Immunostaining of NDN protein in CRC tissue samples and normal colorectal tissues. **(B)** Expression analyses of NDN protein in 12 surgical CRC tissues and the paired normal intestine epithelial samples using Western blot. β-tubulin was used as a loading control. **(C)** qRT-PCR analysis of NDN expression in 21 paired colorectal cancer tissues; NDN was quantified relative to the matched adjacent no tumor tissues. Error bars represent means ± SD calculated from three parallel experiments. **(D)** Expression analyses of NDN protein and mRNA in the normal intestinal epithelial cell FHC and five CRC cell lines through Western blot and qRT-PCR. Each bar represents the mean ± SD of three parallel replicates. **(E)** Influence of NDN expression on overall survival through Kaplan-Meier analysis in 84 CRC patients.

Subsequently, the expression level of NDN was examined in 84 cases of paraffin-embedded CRC tissue sections using Immunohistochemical (IHC) assays. The result showed that NDN protein located both in the cytoplasm and nucleus (Figure 1A). In agreement with the analytical results from the published CRC microarray dataset, the markedly decreased expression of NDN was observed in 65.5% (55/84) CRC tumor tissues (Figure 1A right) compared with that in adjacent non-tumor tissues (Figure 1A left). And the low expression level of NDN protein was significantly correlated with poor differentiation and advanced TNM stage (*p*<0.05) (Table 1). The results of the Kaplan-Meier survival analysis indicated that patients with low NDN expression levels had a poor prognosis (Figure 1E). Cox regression analyses revealed that NDN expression and TNM stage were recognized as independent prognostic factors in this study (Supplementary Table 2).

**Table 1:** The relationship between the expression of NDN and clinicopathological parameters

Clinicopathological variables	NDN expression		Z	P value
	Low	High		
Age				
≤Mean(64)	15	20	−1.100	0.271
>Mean(64)	27	22		
Gender				
Male	22	23	−0.217	0.828
Female	20	19		
Differentiation				
Well	2	5	−2.526	0.012
Moderate	27	33		
Poor	13	4		
TNM classification				
I-II	11	21	−2.233	0.026
III-IV	31	21		

A decrease in NDN expression was also observed in surgically resected CRC tissues (T) compared with their matched non-tumor tissues (N) using Western blot and qRT-PCR analyses (Figure 1B, 1C). Furthermore, the expression of NDN was detected in the normal intestinal epithelial cell line FHC and five CRC cell lines SW480, HT29, HCT116, LS174T and RKO using Western blot and qRT-PCR analyses. The results indicated that the level of NDN was significantly lower in all the five CRC cell lines compared to the levels in the FHC cells (Figure 1D).

### Overexpression of NDN attenuated CRC cell proliferation through cell cycle G1 arrest *in vitro* and restrained tumorigenesis *in vivo*

**Figure 2:**
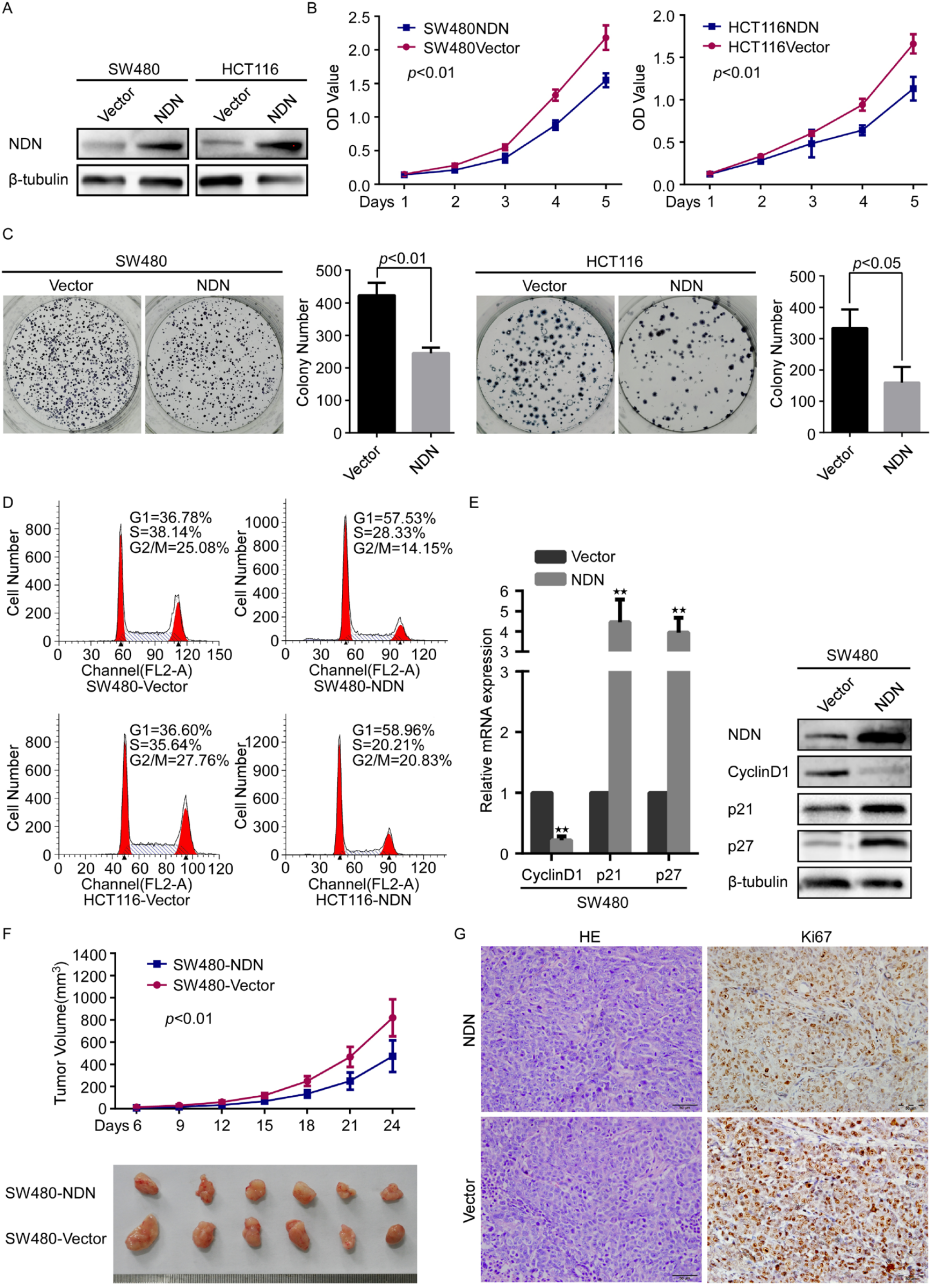
Overexpression of NDN attenuated CRC cell proliferation through cell cycle G1 arrest *in vitro* and reduces tumorigenesis *in vivo* **(A)** Overexpression of NDN in SW480 and HCT116 cells analyzed through Western blot. β- tubulin was used as a loading control. **(B and C)** The over-expression of NDN inhibits SW480 and HCT29 cell proliferation in CCK8 cell proliferation assays **(B)** and colony formation assays **(C)**. **(D)** Flow-cytometry analysis of the cell cycle in the indicated CRC cells. **(E)** qRT-PCR and Western blot analyses of CyclinD1, p21 and p27 in the indicated cells. **(F)** The xenograft models were generated after injecting SW480/Vector and SW480/NDN cells in nude mice (n = 6/group). The tumor volumes were measured on the indicated days. The data points represent the mean tumor volumes ± SD. **(G)** The sections of tumor were subjected to H&E staining or IHC staining using an antibody against Ki-67. Error bars represent the means ± SD from three independent experiments.

NDN was stably expressed in SW480 and HCT116 cells (Figure 2A). CCK8 cell proliferation and colony formation assays revealed that the over-expression of NDN significantly attenuated the growth rate of SW480 and HCT116 cells compared with their control cells (Figure 2B, 2C).

The distribution of cells within the stages of the cell cycle was determined using flow cytometry assay. NDN over-expression significantly increased the proportion of cells in the G1/G0 phase and decreased the proportion of cells in the S and G2/M phase (*p*<0.05) (Figure 2D). Additionally, significant increases in the expression of p21 and p27, and decreases in CyclinD1 were observed in NDN-over-expressing cells (Figure 2E).

Consistent with *in vitro* observations, the subcutaneous tumors generated from SW480-NDN cells were smaller than those derived from SW480-Vector cells (Figure 2F, Supplementary Figure 3). IHC staining confirmed that the tumors formed by SW480-NDN cells showed a lower Ki-67 index than tumors from the control group (Figure 2G).

### Inhibition of NDN promotes CRC progression *in vitro* and *in vivo*

**Figure 3:**
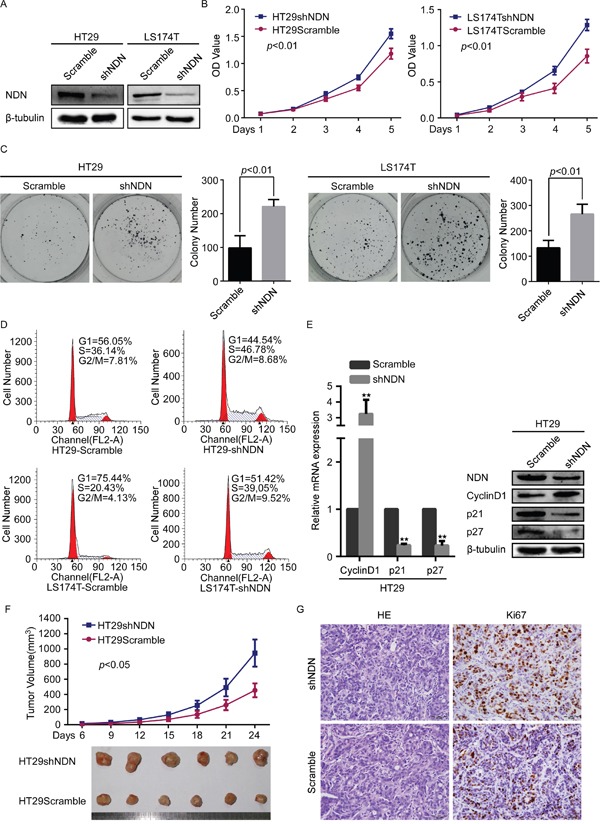
Depletion of NDN promotes CRC progression *in vitro* and *in vivo* **(A)** Silencing of NDN in shRNA-transduced stable HT29 and LS174T cells. β- tubulin was used as a loading control. **(B and C)** Reduction of endogenous NDN promoted cell growth in CCK8 cell proliferation assays **(B)** and colony formation assays **(C)**. **(D)** Flow-cytometry analysis of the cell cycle in the indicated CRC cells. **(E)** qRT-PCR and Western blot analyses of CyclinD1, p21 and p27 in the indicated cells. **(F)** The xenograft model was generated after injecting the HT29/Scramble and HT29/shNDN cells in nude mice (n = 6/group). The tumor volumes were measured on the indicated days. Data points represent the mean tumor volumes ± SD. **(G)** The sections of tumor were subjected to H&E staining or IHC staining using an antibody against Ki-67. Error bars represent the means ± SD from three independent experiments.

To further evaluate the potential effect of NDN on the proliferation and tumorigenesis in CRC, endogenous NDN was suppressed using shRNA specifically targeting NDN in HT29 and LS174T (Figure 3A). The silencing of NDN obviously promoted the growth of HT29 and LS174T cells compared to the scramble transfected cells (Figure 3B, 3C).

Flow cytometry analysis confirmed that the depletion of NDN evidently reduced the percentage of cells in the G1/G0 phase and increased the percentage of cells in the S and G2/M peak (*p*<0.05) (Figure 3D). As shown in (Figure 3E), CyclinD1 was significantly up-regulated, whereas p21, p27 were strikingly down-regulated in NDN-inhibiting cells.

Subcutaneous tumorigenesis assays exhibited that the tumors in the sh-NDN group grew much faster than those in the control group (Figure 3F, Supplementary Figure 3). Immunohistochemical staining substantiated that the cell proliferation index Ki-67 was increased in the sh-NDN group compared with the control group (Figure 3G).

### NDN directly binds to the promoter of LRP6, suppresses its expression and inhibits the canonical Wnt signaling pathway

**Figure 4:**
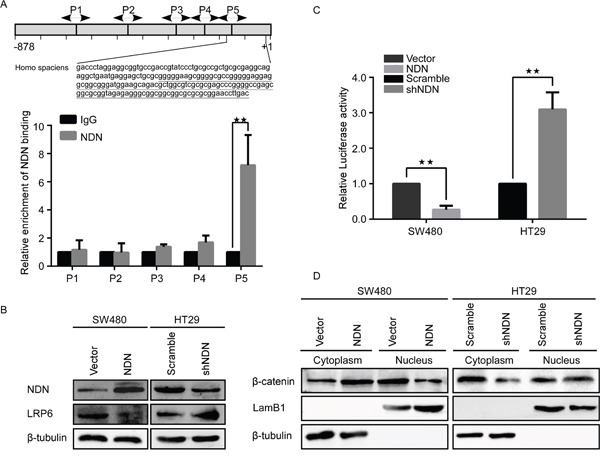
NDN directly binds to the GN box in the promoter of LRP6 in CRC cells and suppresses the Wnt signaling pathway **(A)** ChIP assay showed a potential binding region (5F to 5R, -190bp to 0bp) of NDN in LRP6 promoter. **(B)** Expression of LRP6 in the indicated cells. **(C)** The Wnt signaling luciferase reporter assay of the indicated cells transfected with NDN or shNDN vector, respectively. **(D)** Western blot assay for β-catenin in cytoplasm and nucleus of the indicated cells. LamB1 and β- tubulin served as loading controls for nuclear and cytoplasmic proteins. Error bars represent the means ± SD from three independent experiments, * *p*<0.05, ** *p*<0.01.

Previous studies have reported that NDN is a transcription repressor that directly binds to DNA [15] or indirectly regulates transcription through interactions with other transcription factors such as E2F1 and p53 [16, 25]. The specific target genes of Ndn have been identified by ChIP-sequencing in cells stably over-expressing HA tagged wild-type Ndn [12]. Among these genes, LRP6 promotes cell proliferation through the Wnt signaling pathway in CRC [11]. Bioinformatics analysis revealed NDN contains a putative binding site in the promoter of LRP6 gene at -190 to 0 bp (GN box). ChIP analysis using five pairs of primers covering −878 to +10 bp of the LRP6 promoter (Supplementary Table 5) showed that NDN binds to the specific promoter (−190 to 0 bp) of LRP6 (Figure 4A). The up-regulation of NDN decreased the expression of LRP6 in SW480 cells, however, the reduction of NDN in HT29 cells yielded an opposite effect (Figure 4B).

The results of the Wnt signaling luciferase reporter assays showed that the ectopic expression of NDN remarkably decreased the luciferase activity of Wnt, while the inhibition of NDN showed an increase in the luciferase activity compared to the control group (Figure 4C). In addition, Western blot analysis indicated that over-expression of NDN decreased the intranuclear β-catenin protein (Figure 4D).

### Inhibition of LRP6 reduces the cell proliferation induced by the knockdown of NDN in CRC

**Figure 5:**
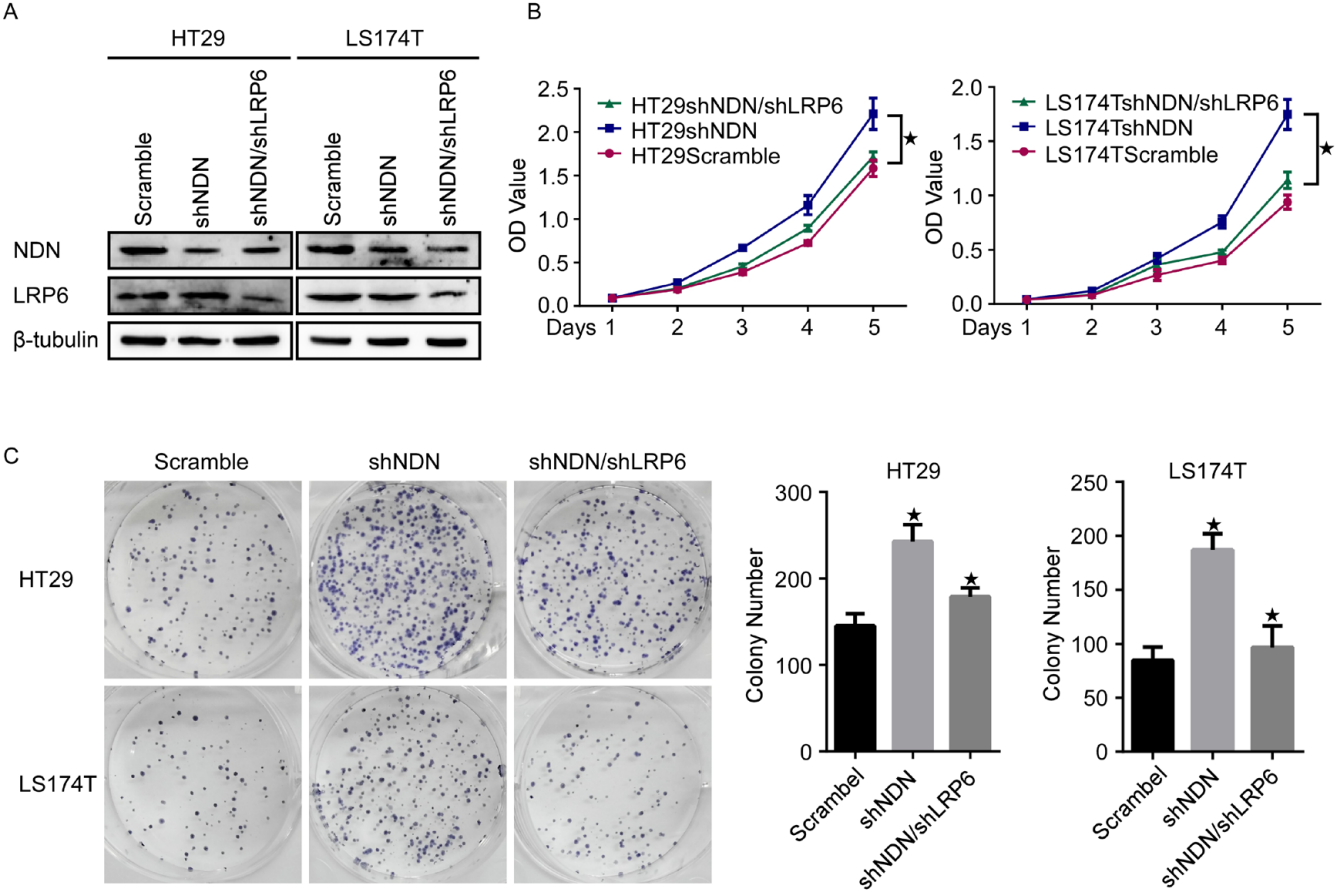
Inhibition of LRP6 reduces the CRC cell proliferation induced through the knockdown of NDN **(A)** LRP6 was down-regulated in HT29-shNDN and LS174T-shNDN cells. **(B and C)** Inhibition of LRP6 suppressed the cell growth resulting from the down-regulation of NDN in CCK8 cell proliferation assays **(B)** and colony formation assays **(C)**. Error bars represent the means ± SD from three independent experiments, * *p*<0.05.

We next inhibited the expression of LRP6 in HT29shNDN and LS174TshNDN cells (Figure 5A) and investigate whether the proliferation of NDN knockdown cells changed using CCK8 cell proliferating assays and colony formation assays. The results revealed that inhibition of LRP6 after the knockdown of NDN reduced the cell proliferation compared to the knockdown of NDN alone in CRC cells (Figure 5B, 5C).

### NDN is hypermethylated in CRC

**Figure 6:**
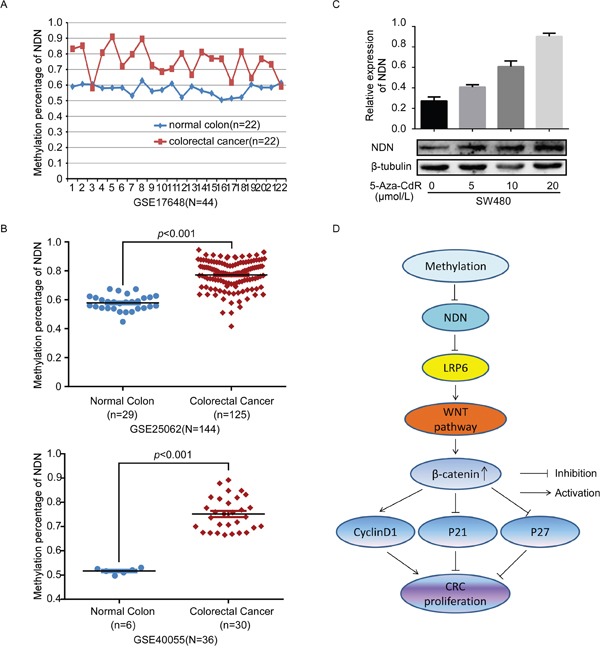
NDN is hypermethylated in CRC **(A and B)** The results of GEO analysis of the methylation level in the promoter of NDN in CRC compared with that in the normal intestinal mucosa. **(C)** Treatment with a DNA demethylation agent 5-Aza-CdR increased NDN expression at different levels in the CRC cell line SW480. **(D)** Proposed model: promoter hypermethylation suppressed the NDN expression, however, NDN down-regulated LRP6 and attenuated the Wnt signaling pathway, and eventually suppressed the proliferation of CRC.

**Figure 7:**
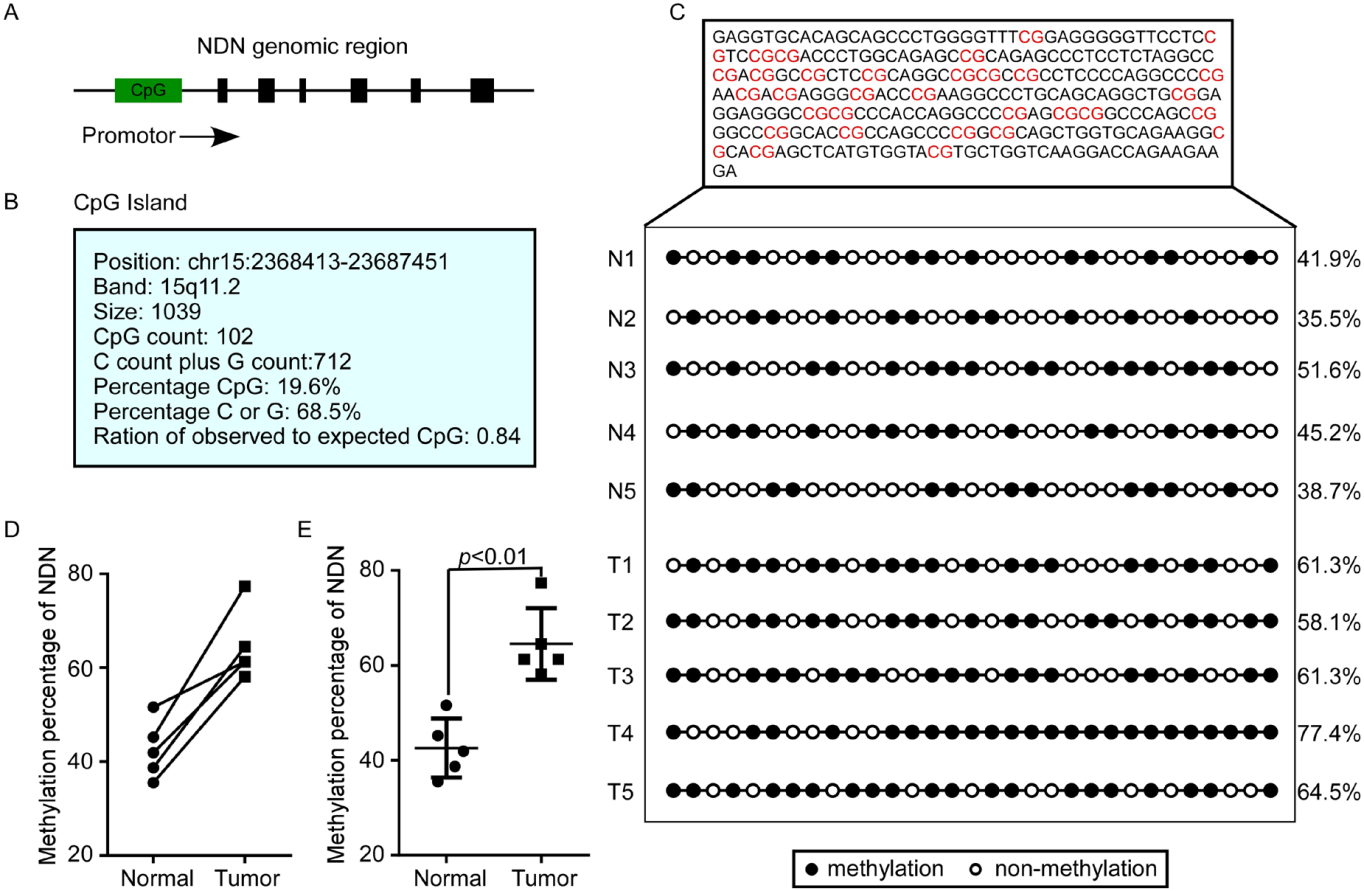
The NDN CpG island was hypermethylated in CRC tissues compared to the normal tissues **(A and B)** The CpG island in the promoter of NDN. **(C, D and E)** The detection of the degree of CpG island methylation in CRC tissues.

The loss of gene expression reflected deletion mutations, epigenetic alterations and post-transcriptional regulation. We analyzed the mutation of NDN in CRC using cBioPortal for Cancer Genomics (http://www.cbioportal.org/), and observed no significant CNV (copy, number, variation) of NDN in CRC (Supplementary Figure 2). However, the data obtained from the GEO analysis indicated that hypermethylation in the promoter of NDN exits in CRC tissues compared to the normal colon mucosa (*p*<0.001) (Figure 6A, 6B). In consistent with GEO analysis, the NDN CpG islands were hypermethylated in CRC tissues compared to the normal tissues using bisulfite sequencing PCR (BSP) (Figure 7). The average methylation percentage of CpG in CRC tissues (64.52 ±3.375%) was significantly higher than that in the corresponding normal tissues (42.58±2.774%; *p*<0.01) (Figure 7D, 7E). Moreover, the NDN expression level increased and the luciferase activity of Wnt decreased in a dose-dependent manner in SW480 cells treated with the demethylating agent 5-Aza-CdR (Figure 6C, Supplementary Figure 4). Thus, the hypermethylation of the NDN promoter leads to NDN gene silencing.

## DISCUSSION

CRC is a common digestive tract tumor, and the progression of CRC is a multistep process involves several genetic changes or epigenetic modifications. The NDN gene encodes the 321 amino acid protein necdin in human, and is widely expressed in normal tissues including bladder, brain, colon and liver [17]. Oncomine (https://www.oncomine.org/resource/login.html) analysis [26] showed that the expression of NDN is down-regulated in multiple tumor types (e.g., bladder, breast, ovarian and colorectal cancer) compared with normal tissues. Indeed, NDN was down-regulated in urothelial carcinomas, promoted anoikis, repressed colony formation and anchorage-independent growth [17, 27]. The loss of NDN expression also promotes cancer cell motility, and invasion in ovarian cancer [18]. These evidences indicated that NDN might be a tumor suppressor.

Analysis of GEO CRC microarray dataset revealed that NDN was markedly down-regulated in primary CRC tissues compared to normal colon tissues. In accordance with the analysis, the current study also showed that NDN was down-regulated in human CRC. The reduced expression of NDN was associated with poor differentiation, advanced TNM stage and poor prognosis of CRC. Furthermore, the over-expression of NDN inhibited cell proliferation *in vitro* and tumor growth *in vivo* by leading cell cycle arrest. These data support the conclusion that NDN may function as a tumor suppressor in CRC.

The vast majority of published studies on the NDN have focused on the expression phenotypes and biological functions. However, the loss of NDN expression and the role of NDN in the development and progression of CRC remain unclear. The result of GEO CRC microarray dataset analysis suggested that the promoter of NDN was hypermethylated compared to the normal colon mucosa. Increasing studies showed that the monoallelic expression of an imprinted gene could be established and maintained through differential DNA methylation between the parental alleles [28, 29]. DNA hypermethylation was markedly associated with the initiation and progression of various types of tumors [30–34]. NDN is a maternally imprinted gene, and it has been identified that tumor-specific hypermethylation in the key CpG islands was correlated with reduced expression of NDN in primary urothelial carcinoma and ovarian cancer [17, 18]. Our study revealed that the CpG islands in the promoter of NDN are hypermethylated in CRC tissues compared to the corresponding normal colorectal tissues, and the loss expression of NDN was associated with the degree of promoter hypermethylation in CRC. The results may provide references for the studies of NDN down-regulation in other tumors. However, the methylation status of the same gene may be different in different types of tumors, so it still needs further investigations to explore the methylation status of NDN in other types of tumors.

It has been reported that NDN interacts with the transactivation domain of E2F1 and suppresses E2F1-dependent transactivation [16]. Moreover, NDN recognizes guanosine(G)-rich sequences that encompass multiple G clusters and intervening mono- or di-nucleotide of A, T and C, referred to as the GN box and directly serves as a transcriptional repressor [15]. We detected a GN box in the promoter of LRP6 and demonstrated that NDN directly binds to the GN box in the promoter of LRP6 to inhibit the expression of LRP6.

LRP6 is a member of the low-density lipoprotein receptor family, and acts as a co-receptor for Wnt ligands, which interacts with the seven transmembrane receptor of the Frizzled (Fzd) family and activates the canonical Wnt/β-catenin signaling pathway [35–37]. The present results showed that the over-expression of NDN attenuated the Wnt/β-catenin signaling activities, and decreased the intranuclear β-catenin expression through the suppression of LRP6 transcription after directly binding to its GN box in the promoter.

In summary, these findings suggest that the promoter hypermethylation leading to the loss of NDN gene expression occurs in CRC. The down-regulation of NDN is associated with poor differentiation, late TNM stages and worsened overall survival in patients with CRC. NDN affects tumor cell proliferation in CRC by inhibiting the expression of LRP6, which is a key factor in the activation of the Wnt signaling pathway. Restoration of NDN might represent a useful therapeutic approach for targeting malignant CRC.

## MATERIALS AND METHODS

### Tissue specimens

The clinical research was performed according to the written approval obtained from the Southern Medical University Institutional Board (Guangzhou, China). All specimens were collected with the informed consent of patients. CRC tissues and matched adjacent normal mucosa samples were collected from Nanfang Hospital, Southern Medical University from 2012 to 2015, including paraffin-embedded tissues (n=84) and fresh surgical specimens (n=21). The surgically resected tissue samples were immediately frozen in liquid nitrogen until future analysis. The medical records of patients were reviewed for the acquisition of the clinicopathological information: age, gender, differentiation, and TNM stage. Survival data were available for the cohort of 84 patients. The median follow-up time was 47.36 (range, 1-122.7) months.

### Cell culture

Human normal intestinal epithelial cell FHC and five CRC cell lines SW480, HT29, HCT116, LS174T, and RKO were obtained from the The Global Bioresource Center (ATCC, USA). The cells were cultured in RPMI1640 (Gibco, Grand Island, NY, USA) supplemented with 10% fetal bovine serum (FBS) (Gibco, Grand Island, NY, USA) at 37°C in a humidified atmosphere with 5% CO_2_.

### Immunohistochemistry (IHC)

The 3 μm-thick tissue sections were deparaffinized and rehydrated, and incubated with primary antibody at 4°C overnight. Prior to incubation with anti-NDN (1:300 dilution; Abcam, Cambridge, MA, USA), and anti-Ki67 antibodies (1:100 dilution; Bioworld Technology Inc., St. Louis Park, MN, USA), the sections were heated in 0.01M sodium citrate buffer, pH6.0 for antigen retrieval, and incubated in 3% H_2_O_2_ to inhibit endogenous peroxidase activity. On the next day, the sections were washed and incubated with the secondary antibody for 30 minutes at room temperature. Finally, the slides were developed using a DAB chromogen kit and counterstained with Mayer's hematoxylin.

The total NDN and Ki67 immunostaining score was calculated as the sum of the percentage positivity of stained tumor cells and the staining intensity. The percentage positivity was scored from 0 to 3, with 0 for<10%, 1 for 10–30%, 2 for 31–50%, and 3 for>50%. The staining intensity was scored from 0 to 3, with 0 for no staining, 1 for weakly stained, 2 for moderately stained, and 3 for strongly stained. Both the percentage positivity of cells and staining intensity were determined in a double-blinded manner. Subsequently, NDN and Ki67 expression was calculated as the value of percentage positivity score × staining intensity score, which ranged from 0 to 9. The final expression level of NDN and Ki67 was defined as ‘low’ (0–4) and ‘high’ (5–9).

### RNA extraction and quantitative reverse transcription PCR (qRT-PCR)

RNA extraction and qRT-PCR were performed as previously described [38]. The primer sequences used for qRT-PCR are listed in Supplementary Table 1.

### Western blot assay (WB)

Protein lysates were prepared using a lysis buffer and quantified using a bicinchoninic acid (BCA) protein quantification kit (KeyGen Biotech, China). The proteins were separated using 10% SDS-PAGE and transferred onto a PVDF membrane and blotted according to standard methods. The membrane was subsequently blocked in PBST solution containing 5% non-fat milk and incubated at 4°C overnight with specific antibodies anti-NDN (1:1000 dilution; Abcam, Cambridge, MA, USA), anti-LRP6 (1:1000 dilution; Abcam, Cambridge, MA, USA), anti-CyclinD1 (1:500 dilution; Proteintech, USA), anti-p21 (1:500 dilution; Proteintech, USA), anti-p27 (1:500 dilution; Proteintech, USA), anti-LamB1 (1:1000 dilution; Proteintech, USA) and anti-β-tubulin (1:1000 dilution; Sigma, Saint Louis, MO, USA), followed by incubation with their respective second antibodies. Subsequently, the membranes were detected using Pierce ECL Western Blotting Substrate (Thermo Scientific, USA).

### Construction of plasmids and transfection

To generate a NDN expression vector, the full-length human NDN cDNA was PCR-amplified and cloned into the lentiviral vector psin-EF2 (deposited in Southern Medical University). The human shRNA sequences specifically targeting NDN and LRP6 were cloned into pLVTHM (deposited in Southern Medical University). The primers used to generate the NDN construct are listed in Supplementary Table 3, and the shRNA nucleotide sequences for repressing NDN and LRP6 are listed in Supplementary Table 4. HEK293T cells were used as a packaging cell line. Recombinant lentiviruses were produced through the transient transfection of HEK293T cells using the calcium phosphate method, and the transfected cells were selected in medium containing 1 μg/ml puromycin.

### CCK-8 cell proliferation assay

Cell proliferation rates were measured using Cell Counting Kit-8 (CCK-8) (Dojindo Laboratories, Japan). Indicated cells were seeded at 1×10^3^ per well in 96-well plate. The cell proliferation assay was performed on days 1, 2, 3, 4, and 5. 10 ml CCK-8 reagent was added to each well and then the plate was incubated for 2 h at 37°C. At the endpoint of incubation, the absorbance was measured at 450 nm using a Vmax microplate spectrophotometer (Molecular Devices, Sunnyvale, CA). Each sample was assayed in triplicate and repeated 3 times independently.

### Colony formation and flow cytometry assays

The plated colony formation, flow cytometry assays were performed as previously described [38, 39].

### TOP-Flash Wnt reporter

The activity of the Wnt pathway was examined using a TOP-Flash luciferase reporter. The indicated cells were co-transfected with 1.5 μg TOP-FLASH or FOP-FLASH and 0.15 μg pRL-SV40 plasmid. Luciferase activity was measured with the Dual-Luciferase Reporter Assay System (Promega); the ratios of TOP/FOP were calculated and used as indicators of Wnt signaling activity.

### Xenograft growth assay

To generate xenograft tumors, transfected CRC cells (2×10^6^) together with their control groups were subcutaneously injected into the hind limbs of female athymic BALB/c nude mice between 4 and 6 weeks of age (n = 6 per group). The nude mice were maintained in laminar flow cabinets under specific pathogen-free conditions. All experiments were conducted according to the national guidelines and approved through the ethical committee of the Southern Medical University. The primary tumor growth was measured using a slide caliper and the tumor volume was determined using the formula (length × width^2^)/2 [40]. After euthanasia, the tumors were surgically removed, fixed in formalin (neutral buffered 10%), embedded in paraffin, and prepared into 3-μm sections for staining with hematoxylin and eosin (H&E).

### Chromatin immunoprecipitation (ChIP)

A ChIP assay was performed according to the EZ-ChIP assay kit (Millipore, Temecula, CA, USA). Briefly, 1 × 10^6^ cells were lysed using sodium dodecyl sulfate lysis buffer, and sheared to lengths ranging from 200 to 1000 bp using sonication. NDN or IgG antibody was used to precipitate the DNA-protein complex and subsequently elute the DNA from the antibody. The immunoprecipitated DNA was examined using qPCR. The five pairs of primers used for qPCR analysis are listed in Supplementary Table 5.

### Bisulfite modification and genomic sequencing

The methylation status of the CpG dinucleotides in the NDN promoter was analyzed. A bisulfate-sequencing assay was performed using 1.0 mg of bisulfite-treated genomic DNA from the clinical samples. Bisulfite conversion was performed using the EpiTect Bisulfite Kit (QIAGEN, German) according to the manufacturer's instructions. The fragments of interest were amplified using the following specific primer pairs designed with the MethPrimer software47: forward, 5′- GAGGTGTATAGTAGTTTTGGGGTTT -3′; reverse, 5′- TCTTCTTCTAATCCTTAACCAACAC -3′. The PCR products were gel purified and cloned into the pMD19-T vectors using pMD19-T vector cloning kit (TAKARA, Japan). Individual bacterial colonies were picked and sequenced to analyze DNA methylation.

### Statistical analysis

Statistical analyses were performed using SPSS13.0 for windows. The data were presented as the means ± SD in at least 3 independent experiments. The differences between groups were examined using one-way ANOVA or two-tailed Student's t-test. The relationships between NDN expression and clinicopathological characteristics were determined using the Mann-Whitney U-test. The survival curve was plotted using the Kaplan–Meier method and compared using the log-rank test. A value of *p*<0.05 was considered statistically significant.

### Accession numbers for data sets

The different expression of NDN generated in the study came from the GEO database (GSE41258). The methylation percentage of NDN in the normal and colorectal cancer tissues reanalyzed in the study came from the GEO database (GSE17648, GSE25062 and GSE40055).

## SUPPLEMENTARY FIGURES AND TABLES


