# Supplementary material for: Hypermethylation of NDN promotes cell proliferation by activating the Wnt signaling pathway in colorectal cancer
Source: Oncotarget. 2017 May 3;8(28):46191–203. doi: 10.18632/oncotarget.17580 (PMC5542259; doi:10.18632/oncotarget.17580)
Supplement: Supplementary file 1 [file oncotarget-08-46191-s001.pdf]

Hypermethylation of NDN promotes cell proliferation by activating the Wnt signaling pathway in colorectal cancer

SUPPLEMENTARY MATERIALS

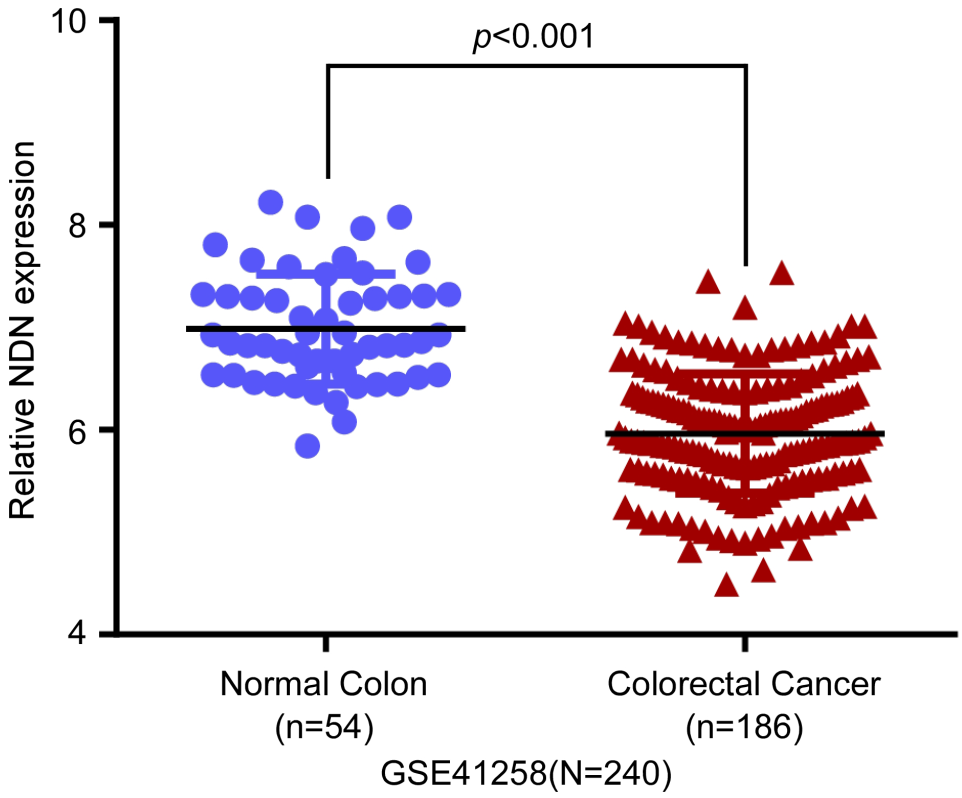

Supplementary Figure 1: Analysis of NDN expression in CRC data from GEO. Analysis of NDN expression in Gene Expression Omnibus (GEO, http://www.ncbi.nlm.nih.gov/geo/) data (GSE41258, n=240).

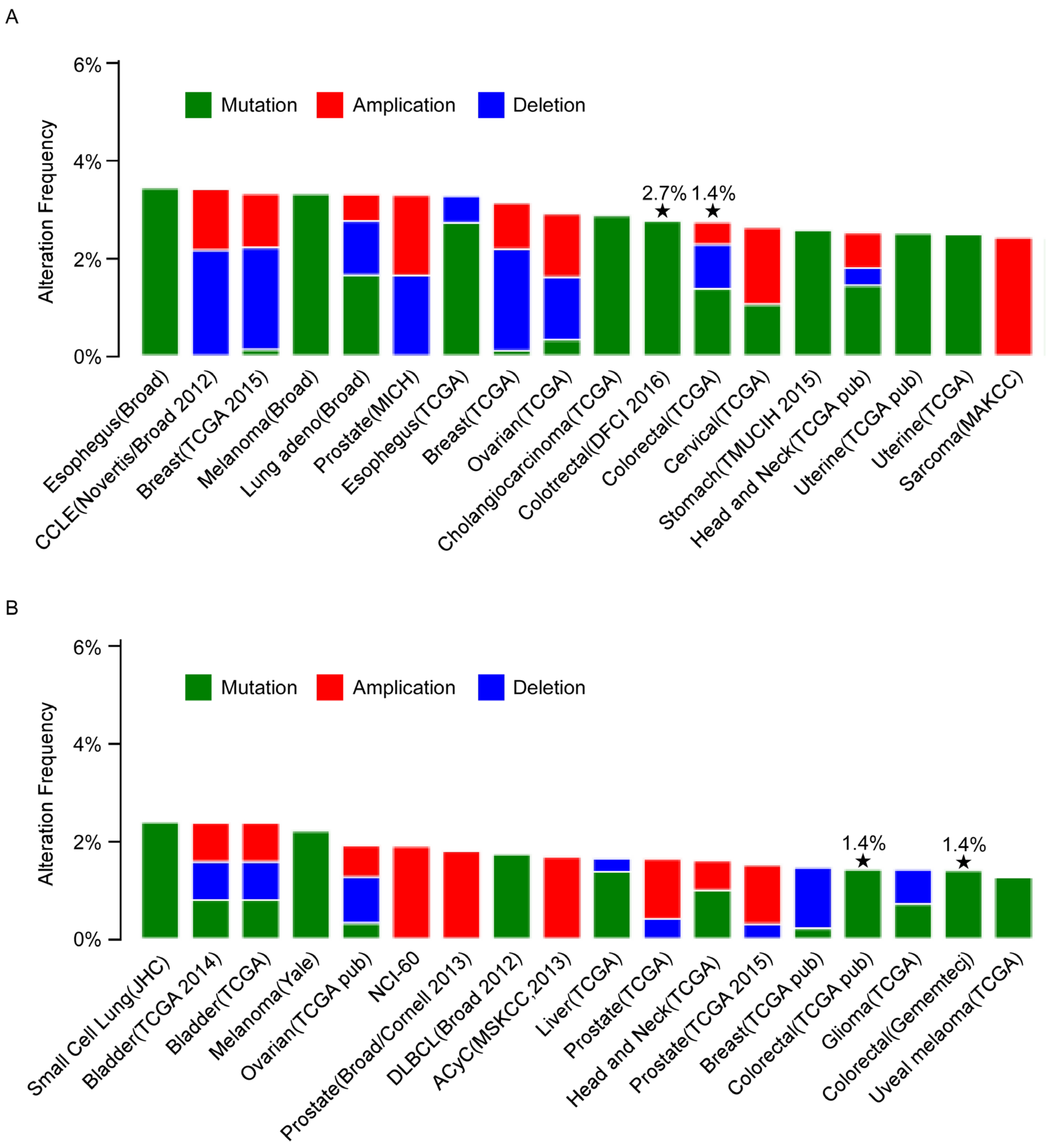

Supplementary Figure 2: Analysis of NDN mutation in CRC using cBioportal for Cancer Genomics. Analysis of NDN mutation in CRC using cBioPortal for Cancer Genomics (http://www.cbioportal.org/).

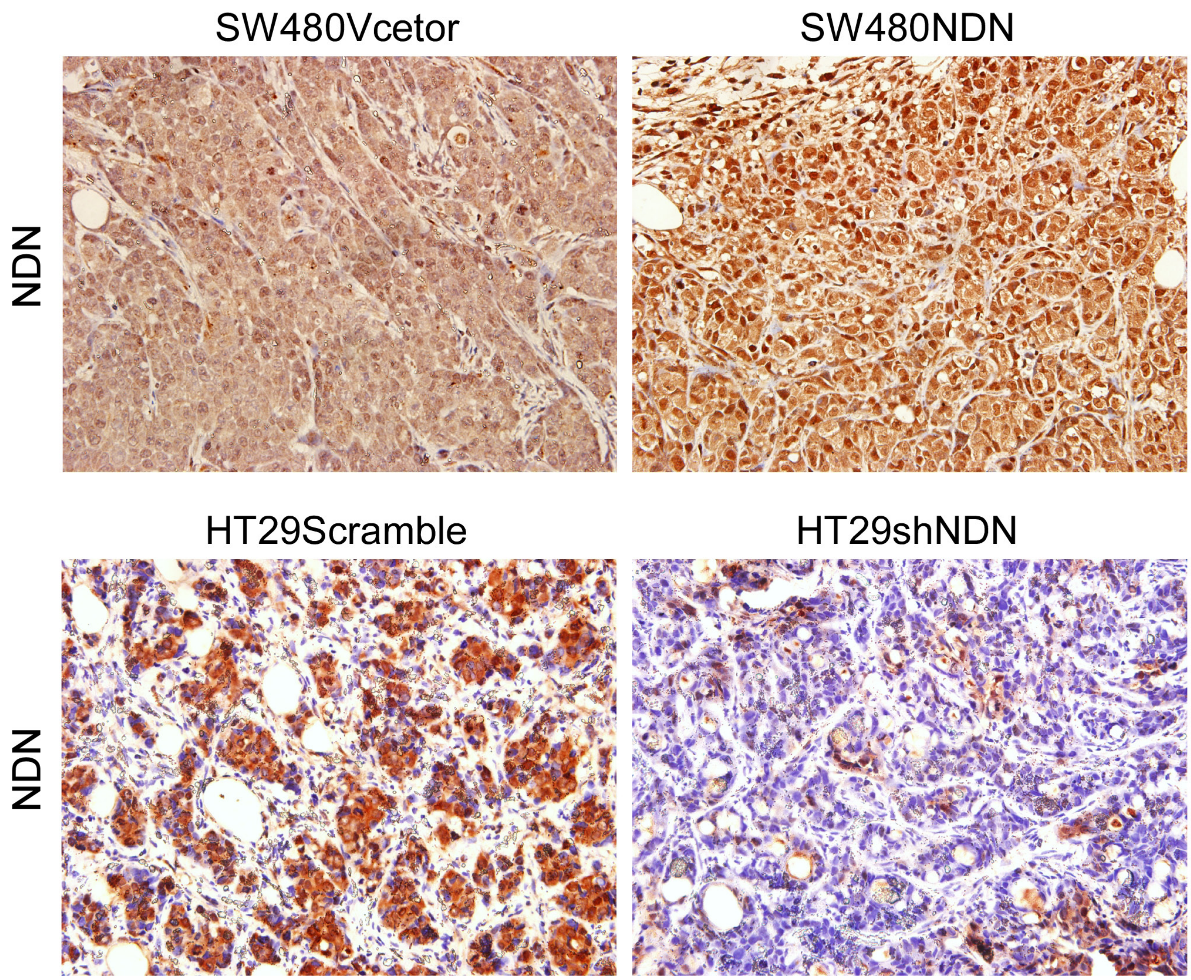

Supplementary Figure 3: NDN expression in the xenograft tumors. NDN expression was tested in the xenograft tumors using IHC.

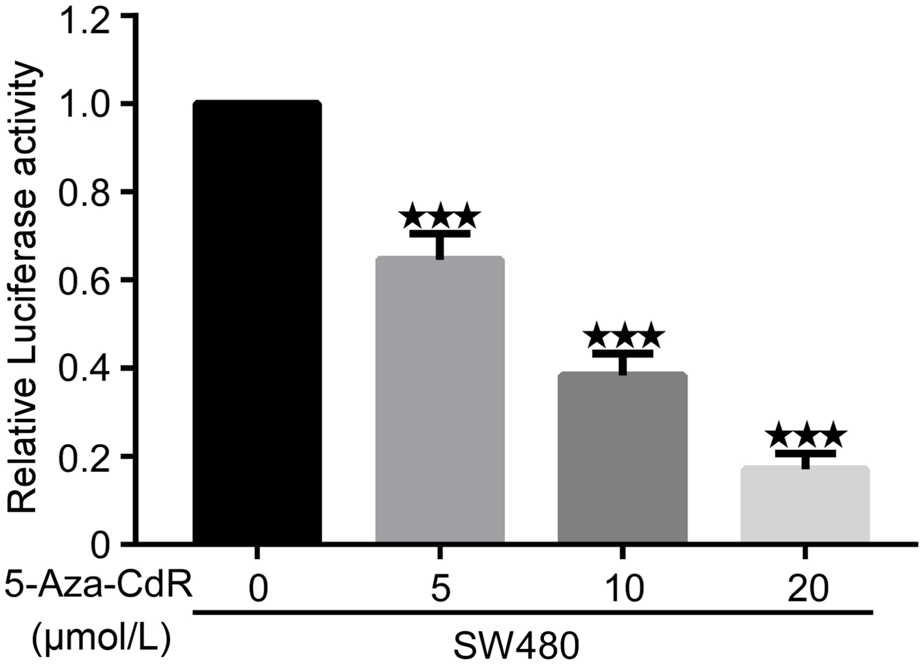

Supplementary Figure 4: The increased demethylation correlates with decreased Wnt signaling in CRC cells. The Wnt signaling luciferase reporter assay of the indicated cells treated with the demethylating agent 5-Aza-CdR.

Supplementary Table 1: Primer sequences used for qRT-PCR (5' to 3')

Gene	Forward primer	Reverse primer
NDN	CGCGAAATCACCAAGATGC	AGACACTGCTGCGAGGGTAG
CyclinD1	GCGAGGAACAGAAGTGCG	TGGAGTTGTCGGTGTAGATGC
p21	TGGCACCTCACCTGCTCTG	CGGCGTTTGGAGTGGTAGAA
p27	GGCTCCGGCTAACTCTGA	TCTTCTGTTCTGTTGGCTCTTT
GAPDH	GACTCATGACCACAGTCCATGC	AGAGGCAGGGATGATGTTCTG

Supplementary Table 2: Multivariate analyses of various prognosis parameters in 84 CRC patients using Cox Regression model

Variable	Category	No. patients	Multivariate analyses		
			P	Relative risk	95%CI
NDN	Low	42	0.048	2.172	1.008-4.679
	High	42			
TNM stage	I-II	32	0.005	0.179	0.054-0.591
	III-IV	52			

SE: standard error; RR: relative risk; CI: confidence interval.

Supplementary Table 3: Primer sequences used for amplification and plasmid construction (5' to 3')

Gene	Forward primer	Reverse primer
NDN(P1)	AGGGAGCGCAGGCTCGAAGA	GCGAAAGCACCAACCAAG
NDN(P2)	CGCGGATCCATGTCAGAACAAAGTAAGGA	CTAGACTAGTCTAGTCCTCAGAGACACTGC

Supplementary Table 4: Sequences used for the knockdown of genes (5' to 3')

Gene	Sequence
NDN shRNA	CGCACGAGCUCAUGUGGUA
LRP6 shRNA	GAGAAUGCUACGAUUGUAGTT

Supplementary Table 5: Primer sequences used for ChIP analysis of the binding between NDN and the LRP6 promoter (5' to 3')

	Forward primer	Reverse primer
Primer 1	ATTGTCCCTTTGCTCTTAGGTT	GCTTTCAGCGTGGATCTGGT
Primer 2	GCCGAGCCGCAGAAAGTA	GGCGAACTGGGAGGGAAA
Primer 3	GCCGGTCCGGGAAAGAAT	AGGGAAGGGAGTCGGCAACC
Primer 4	GGCTTTCCCTCCCAGTTCG	ATACGGTCGGCACCGCCTCCTA
Primer 5	GACCCTAGGAGGCGGTGC	AAAGGGGCACGTCAAGGT
